# Protocol for one-step design of a triple sgRNA-based CRISPR/Cas9 construct for neuronal gene knockout in the mouse brain

**DOI:** 10.1016/j.xpro.2026.104715

**Published:** 2026-07-21

**Authors:** Gibson Dowd, Yuki Ogawa

**Affiliations:** 1 Department of Biological Sciences, McCausland College of Arts and Sciences, University of South Carolina, Columbia, SC 29208, USA

**Keywords:** Molecular Biology, CRISPR, Neuroscience

## Abstract

CRISPR-Cas9 enables genome editing through the expression of Cas9 and single guide RNAs (sgRNAs). Here, we present a protocol for designing and constructing the vector expressing three sgRNAs targeting a single gene in the mouse brain. We describe steps for CRISPR knockout sgRNA design, plasmid construction and verification, animal preparation, and neonatal adeno-associated viral (AAV) vector delivery. We then detail procedures for brain preparation and immunofluorescence-based validation of gene disruption. This protocol enables rapid, one-step assembly of triple-sgRNA expression cassettes.

For complete details on the use and execution of this protocol, please refer to Ogawa et al.^1^

## Highlights

• Procedures for designing three sgRNAs targeting a single gene
• Guidance on one-step cloning of triple sgRNAs into an AAV-based vector
• Instructions for neonatal intracerebroventricular injection of AAV vectors
• Steps for immunofluorescence validation of neuronal gene knockout in brain sections

## Abstract (editor highlights)

Publisher’s note: Undertaking any experimental protocol requires adherence to local institutional guidelines for laboratory safety and ethics.

## Graphical abstract

**Figure:**
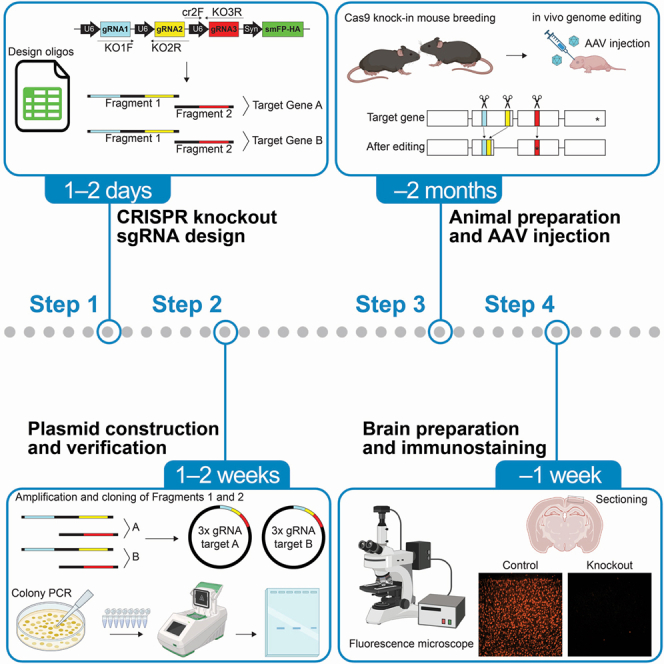

## Teaser

CRISPR-Cas9 enables genome editing through the expression of Cas9 and single guide RNAs (sgRNAs). Here, we present a protocol for designing and constructing the vector expressing three sgRNAs targeting a single gene in the mouse brain. We describe steps for CRISPR knockout sgRNA design, plasmid construction and verification, animal preparation, and neonatal AAV vector delivery. We then detail procedures for brain preparation and immunofluorescence-based validation of gene disruption. This protocol enables rapid, one-step assembly of triple-sgRNA expression cassettes.

## Before you begin

Recent advances in AAV vector technology combined with CRISPR/Cas9 enable efficient postnatal gene disruption in specific tissues, providing a flexible alternative to traditional knockout strategies.^2^^,^^3^^,^^4^ Building on this concept, we developed a streamlined cloning strategy to generate a triple sgRNA expression vector using an AAV vector backbone.

### Innovation

Previous studies have shown that simultaneous targeting of a gene using multiple sgRNAs improves knockout efficiency and reduces dependence on individual sgRNA activity.^5^^,^^6^ However, conventional approaches for multiple sgRNA cloning require sequential assembly of individual sgRNAs into a single vector. Here, we developed a protocol for the simultaneous insertion of three sgRNA sequences in a single cloning step. This method reduces cloning complexity and time. The resulting vectors are compatible with AAV vector production and can be readily applied to both in vitro and in vivo experiments.

### Preparation of oligonucleotides

All oligonucleotides used in this study were purchased from Sigma at a 0.025 μmol scale with desalting purification and supplied in either dried or liquid format. This represents the most cost-effective option from Sigma, and equivalent low-cost options from other vendors (e.g., Azenta/Genewiz) are also suitable.

### Preparation of plasmid templates

The template plasmid “pAAV 3×gRNA KO; Template” is available from Addgene (plasmid #240310), and this protocol is optimized for this backbone.

### Preparation of Cas9 knock-in mice

The Cas9 knock-in mouse line used in this protocol is available from the Jackson Laboratory (RRID: IMSR_JAX:027650). However, other SpCas9 knock-in mouse lines are also theoretically compatible.

### Institutional permissions

All experimental procedures were performed in accordance with the University of South Carolina guidelines. The Institutional Biosafety Committee (IBC) protocol number: 1-0106-0225. The Institutional Animal Care and Use Committee (IACUC) protocol number: 2763-101966-042125. Users of the protocol must obtain similar permissions from their respective institutions.

## Key resources table

**Table:** 

REAGENT or RESOURCE	SOURCE	IDENTIFIER
**Antibodies**		

Anti-NeuN (1:500 dilution)	Millipore	MAB377 (RRID: AB_2298772)
Anti-GFP (1:1,000 dilution)	Thermo	A11122 (RRID: AB_221569)
Goat anti-Mouse IgG2a Alexa Fluor 594 (1:1,000 dilution)	Thermo	A21130 (RRID: AB_1500822)
Goat anti-Rabbit IgG Alexa Fluor 488 (1:1,000 dilution)	Thermo	A11034 (RRID: AB_1500825)

**Bacterial and virus strains**		

NEB stable	NEB	C3040H

**Chemicals, peptides, and recombinant proteins**		

Ampicillin sodium Salt	Fisher	BP176025
LB agar	Sigma	L3027-1kg
LB Broth	Sigma	L3522-1KG
Paraformaldehyde (PFA)	Sigma	441244-1KG
BbsI-HF restriction enzyme	NEB	R3539L
In-Fusion Snap Assembly Master Mix	TAKARA	638949
Gelatin	Sigma	G9382-100g
Vectashield plus	Thermo	NC1864755
Triton-X100	Sigma	T8787-100ml
Hoechst	Thermo	h3569
NaCl	Sigma	S3014-1KG
KCl	Sigma	P5405-500g
NaH_2_PO_4_	Sigma	S9638-500g
Na_2_HPO_4_	Sigma	S7907-500G
D-Sucrose	Fisher	BP220-212
Goat serum	Thermo	16210072
Agarose	Thermo	BP1356500
CloneAmp HiFi PCR Premix	TAKARA	639298
OneTaq 2× Master Mix	NEB	M0482L
OCT compound	Andwin Scientific	14-373-65
PBS	Corning	MT21031CV

**Critical commercial assays**		

NucleoSpin Plasmid Transfection-grade	TAKARA	740490.250
Nucleospin Gel PCR	TAKARA	740609.250

**Experimental models: Organisms/strains**		

spCas9 knock-in mouse (Mus musculus, both sexes)	Jackson Labs	RRID: IMSR_JAX:027650

**Oligonucleotides**		

mRbfox3_KO1F: agaggccatgtttatgTTGGGCTGC TGCTTCTCCGTgtttaagagctaagctggaaacag (60 bp	Sigma	NA
mRbfox3_KO2R: tgcttagctctcaaacGGGTCATGA CCAATAAGAAGcaacaaggtggttctccaaggg (58 bp)	Sigma	NA
cr2F: gtttgagagctaagcagaaagctgc (25 bp)	Sigma	NA
mRbfox3_KO3R: tgcttagctctgaaacGGGCCGTGCT GTGTATAATAcatgtttctggctttccacaag (58 bp)	Sigma	NA
hSyn 0R: cctggtcctaaaacccacttgcac (24 bp)	Sigma	NA
mU6 R: caacaaggtggttctccaaggg (22 bp)	Sigma	NA

**Recombinant DNA**		

pAAV 3×gRNA KO; Template	Addgene	https://www.addgene.org/240310/
pAAV 3×gRNA KO; Rbfox3	Addgene	https://www.addgene.org/240311/

**Software and algorithms**		

Snapgene (ver. 8.2.2)	Dotmatics	https://www.snapgene.com/
Benchling	Benchling	https://benchling.com/
CRISPOR	NA	https://crispor.gi.ucsc.edu/crispor.py
Zen 3.1	Carl Zeiss MicroImaging	NA
ImageJ (ver. 2.9.0/1.54pp)	NIH	https://imagej.net/ij/

**Other**		

Zeiss microscope	Carl Zeiss MicroImaging	Upright Axio-imager M2 microscope fitted with an apotome 3 attachment for structured illumination
Incu-Shaker Mini	Benchmark	H1001-M
ProFlex PCR Systems	Applied Biosystems	A41182
Nanodrop	Thermo Scientific	134005181P5
Gastight Syringe	Hamilton	7653-01
32-gauge Needle	Hamilton	7803-04
Square cover glass (22× 22 mm)	VWR	48366-227
Heating pad	SnuggleSafe	https://snugglesafeusa.com/
Sterile drape	Med Vet	50-306-9164
Metal plate	LepoHome	NA
Tray	US Acrylic Coastal Plastic	NA
Cotton swab	Puritan	22-029-488
Povidone Iodine	Med Vet	50-283-2277
Ethanol	Decon Labs	71002-512

## Materials and equipment

Prepare all solutions and reagents according to the tables below, and store them under the indicated conditions until use.

• To prepare 50 mL of 4% PFA used for brain fixation, refer to the following table:

**Table:** 4% PFA

Reagent	Final concentration	Amount
PFA	4%	2 g
1x PBS	1×	50 mL
Total	NA	50 mL

**CRITICAL:** Heat PBS to boiling in a microwave (1–2 min) before adding PFA. Add PFA and stir thoroughly using a stir bar, then add 10 M NaOH dropwise until the PFA is fully dissolved. Adjust the pH to 7.0–7.4 (optimal: 7.2) using 1 M HCl and 1 M NaOH.

**CRITICAL:** Store the solution at 4°C until use. Prepare fresh on the day of the experiment whenever possible, and do not use solutions older than 1–2 days.

**CRITICAL:** PFA is toxic. Handle in a chemical fume hood while wearing appropriate personal protective equipment (e.g., gloves, lab coat, face mask, and eye protection).

***Note:*** Commercial 4% PFA solutions are available and may be used; however, we have not tested them, and equivalent results are not guaranteed.

• To prepare 1 L of 0.1 M phosphate buffer (PB) used for brain staining, refer to the following table:

**Table:** 0.1 M PB

Reagent	Final concentration	Amount
NaH_2_PO_4_	0.0217 M	2.6 g
Na_2_HPO_4_	0.0810 M	11.5 g
MilliQ	NA	Up to 1 L
Total	0.1 M	1 L

***Note:*** Stir thoroughly using a stir bar.

**CRITICAL:** Store the solution at 20°C–26°C until use. Do not use solutions older than 3–4 weeks.

• To prepare 1 L of 0.05 M PB used for brain staining, refer to the following table:

**Table:** 0.05 M PB

Reagent	Final concentration	Amount
0.1 M PB	0.05 M	500 mL
MilliQ	NA	Up to 1 L
Total	0.05 M	1 L

***Note:*** Stir thoroughly using a stir bar.

**CRITICAL:** Store the solution at 20°C–26°C until use. Do not use solutions older than 3–4 weeks.

• To prepare 50 mL of PBTGS used for brain staining, refer to the following table:

**Table:** PBTGS

Reagent	Final concentration	Amount
Goat serum	10%	5 mL
TritonX-100 (15%)	0.3%	1 mL
0.1 M PB	0.1 M	44 mL
Total	NA	50 mL

***Note:*** Vortex the solution until it becomes homogeneous.

**CRITICAL:** Store the solution at 4°C until use. Do not use solutions older than 1–2 weeks.

## Step-by-step method details

### One-step cloning of triple sgRNAs for knockout

**Timing: 1–2 weeks**

**Timing: 1–2 days (step 1)**

**Timing: 1–3 days (step 2)**

**Timing: 1–2 days (step 3)**

Here, we describe the workflow for designing three sgRNAs targeting a gene of interest and assembling them into an AAV-based triple-sgRNA vector. This section covers target-region selection, sgRNA selection, oligonucleotide design, and one-step cloning of the knockout construct.

***Note:*** For each gene, three sgRNAs are designed to target distinct coding exons whenever possible. When targeting multiple exons is not feasible, sgRNAs are selected to target three regions spaced as far apart as possible within the gene to maximize the likelihood of generating frameshift mutations or large deletions. The sgRNAs are cloned into AAV-based plasmids. We recommend designing two independent sets of sgRNAs for each target gene (design six sgRNAs in total), as editing efficiencies may vary among sgRNAs. Each set should target distinct regions of the gene (e.g., different exons or non-overlapping loci), allowing independent validation of gene disruption. Using two distinct target regions also helps to minimize the likelihood that observed phenotypes arise from off-target effects. The protocol below describes the specific steps for cloning sgRNAs targeting *Rbfox3*. However, this approach is also applicable to other target genes of interest.

**Figure 1:**
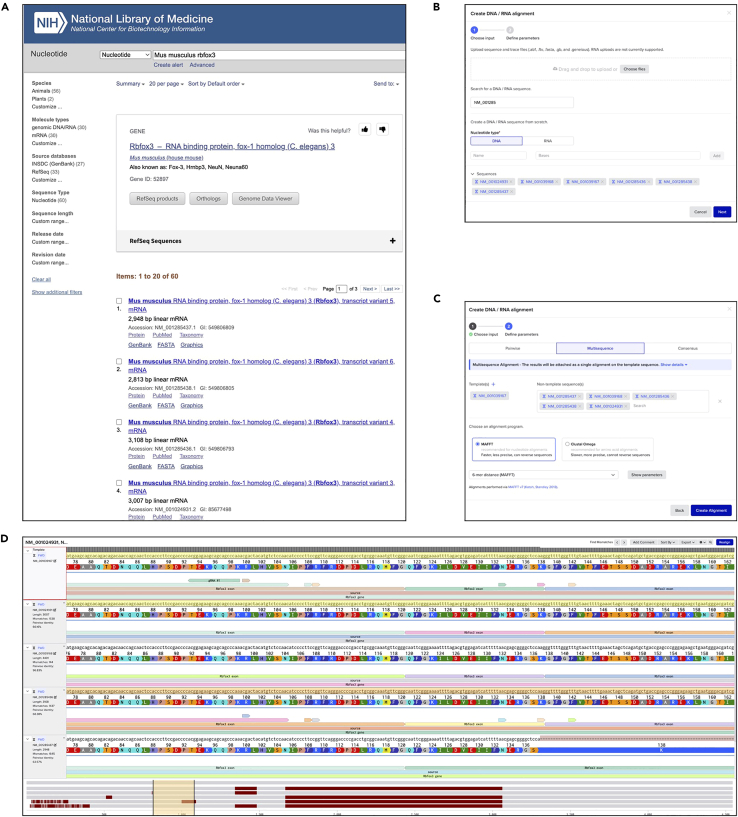
Selection of target nucleotide sequence for CRISPR knockout design (A) To identify optimal sgRNA target sites, the gene of interest (e.g., *Rbfox3*) was searched using the NCBI Nucleotide (https://www.ncbi.nlm.nih.gov/nucleotide). (B and C) Six isoforms were imported into Benchling and aligned using the MAFFT algorithm. (D) In the Benchling user interface, a quick comparison view is shown at the bottom, where conserved sequences are displayed in gray and divergent regions in dark red. The region highlighted in light yellow was selected for downstream analysis in this protocol.
    iii. Download all the available variants.
      ***Note:*** If too many “PREDICTED” isoforms are found, refine the search with “(Mus musculus *Rbfox3*) NOT PREDICTED” to exclude predicted isoforms.
  b. Sequence alignment and selection of constitutive exons for sgRNA design.
    i. Import six *Rbfox3* isoforms into Benchling (https://benchling.com/) (Figure 1B).
      ***Note:*** Any other equivalent software, such as Snapgene, is optimal.
    ii. Align the sequences using multiple sequence alignment (e.g., MAFFT) (Figure 1C).
      ***Note:*** Other alignment software or algorithms can also be used.
    iii. Identify shared constitutive exons across isoforms and select ∼1,000 bp of sequence for sgRNA design (Figure 1D).
      ***Note:*** This selection serves as an initial starting point and does not need to be finalized at this stage. If sgRNA design results are suboptimal, alternative regions can be selected and the process repeated.
      ***Note:*** When feasible, select regions spanning multiple constitutive exons.
      **CRITICAL:** If targeting the first exon, avoid the 5′ UTR and ensure the CRISPR cut site is within the coding sequence.
      **CRITICAL:** Avoid targeting the last exon, as mutations in this region may not induce nonsense-mediated decay and may have a limited impact on gene expression.
      ***Note:*** An example of the selected sequence is shown in Figure 2: ggcaatcaggacgccacggctccacctgaagcgatggcccagccctacccccctgcccagtaccctcctccgcctcagaatggaatcccagctgaatatgccccacctccgcctcatcccacccaggactactccggccagacccctgttccccccgagcacggcatgaccctctacacacccgcacagactcatcctgagcagccaggcactgaggccagcacacagcccattgctgggacccagacggtgccgcaggcagatgaagcagcacagacagacaaccagcaactccacccttccgaccccacggagaagcagcagcccaaacgactacatgtctccaacatccccttccggttcagggaccccgacctgcggcaaatgttcgggcaattcgggaaaattttagacgtggagatcatttttaacgagcggggctccaagggttttgggtttgtaacttttgaaactagctcagatgctgaccgagcccgggagaagctgaatgggacgatcgtagagggacggaaaattgaggtcaataatgccacagcccgggtcatgaccaataagaagcctgggaacccatatgccaatggctggaagctaaaccctgtggtaggaacagtctatgggcctgaattctatgcagtgaccagtttcccctaccccaccacgggcaccgccgttgcctaccggggtgcacacctgcggggccggggccgtgctgtgtataatacatttcgagctgcaccacctccaccccccattccaacttatggagc.

1. CRISPR knockout sgRNA design.
  Here, we describe the procedures for designing three sgRNAs targeting a gene of interest for CRISPR/Cas9-mediated knockout.
  a. Download target sequences
    i. Access NCBI Nucleotide (https://www.ncbi.nlm.nih.gov/nuccore/).
    ii. Search for ‘Mus musculus *Rbfox3’* (Figure 1A).
      ***Note:*** To target other genes, replace Rbfox3 with the gene of interest. The species (Mus musculus) can also be modified as needed.

**Figure 2:**
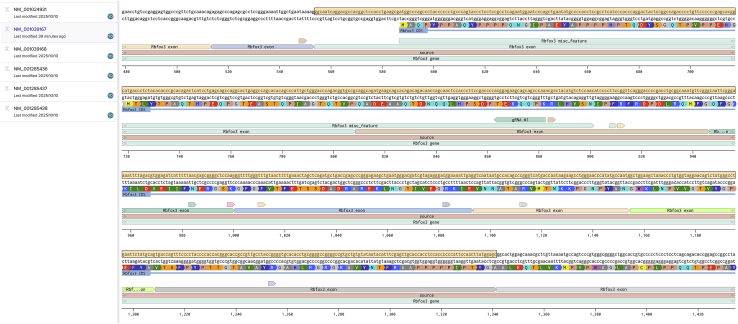
Selection of the constitutive exons for sgRNA design The ∼1,000 bp target region selected in this protocol is shown in one *Rbfox3* isoform (GenBank: NM_001039167). This region spans multiple constitutive exons.
  c. Generate and select sgRNA sequences.
    i. Access the CRISPOR web tool (https://crispor.gi.ucsc.edu/).
      ***Note:*** This tool provides comprehensive scoring for on-target efficiency and off-target potential.
    ii. Insert the target sequence (Figure 3A).
    iii. Select the reference genome.
      ***Note:*** In this example, choose Mus musculus - Mouse UCSC Jun. 2020 (GRCm39/mm39).
    iv. Select the protospacer adjacent motif (PAM) for spCas9.
    v. Submit the query to generate sgRNAs.
    vi. Download the results by selecting the “Guides, all scores” (Figure 3B).

**Figure 3:**
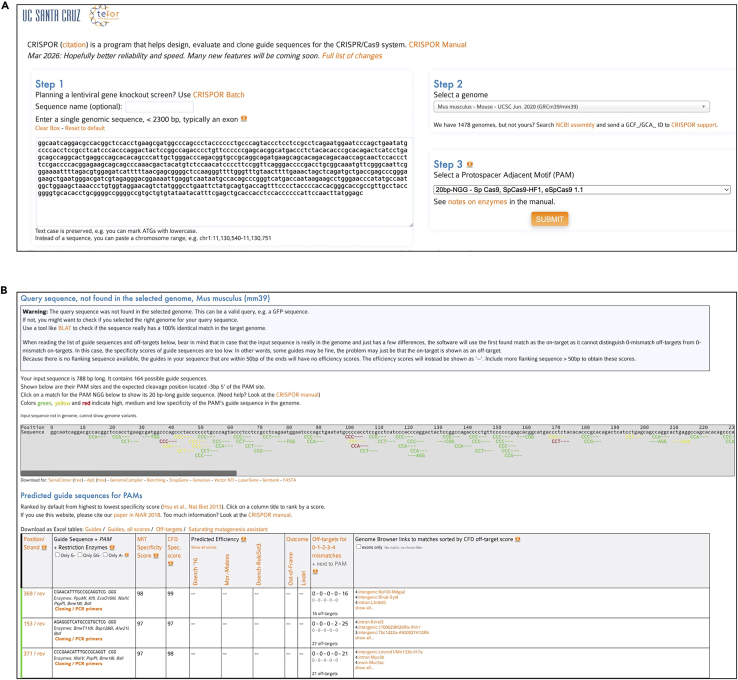
sgRNA design using CRISPOR (A) To identify candidate sgRNAs for CRISPR knockout, the target DNA sequence from constitutive exons was entered into the CRISPOR web tool (https://crispor.gi.ucsc.edu/), and the appropriate reference genome was selected. (B) CRISPOR provides a ranked list of sgRNAs based on predicted on-target efficiency and off-target scores. sgRNA candidates were evaluated based on predicted on-target efficiency, specificity scores, and potential off-target effects.
    vii. Open the downloaded file in Excel. The result is ranked by the MIT specificity scores. The MIT Specificity Score ranges from 0 to 100, with higher scores indicating greater specificity.
    viii. Select the three top-ranked sgRNAs targeting different exons. Here are the examples of the selected sequences without PAM sequences.
          sgRNA #1: TTGGGCTGCTGCTTCTCCGT (MIT score: 69).
          sgRNA #2: CTTCTTATTGGTCATGACCC (MIT score: 81).
          sgRNA #3: TATTATACACAGCACGGCCC (MIT score: 90).
      **CRITICAL:** When possible, select sgRNAs targeting different exons to increase the likelihood of effective gene disruption and reduce the risk of incomplete knockout. In addition, prioritize sgRNAs with high specificity scores to minimize off-target effects.
      **CRITICAL:** Do not select the sgRNA sequences, of which the ‘targetGenomeGeneLocus’ section is empty. It means the sgRNA targets the junction of the exons and is ineffective in cells.
      **CRITICAL:** Do not select the sgRNA sequences noted as ‘Inefficient’ or ‘Not with U6/U3’ on the website. These sgRNAs will not be properly expressed.
      ***Note:*** To generate two independent sgRNA sets (six sgRNAs total), all candidates might be selected from this single region. However, selection depends on CRISPOR results. If CRISPOR analysis yields few suitable sgRNAs in this region, select an alternative region and repeat the analysis.
  d. For subcloning, order oligonucleotides corresponding to the selected sgRNA sequences. The sgRNA sequences are incorporated into forward and reverse primers with additional overhangs for cloning and used for PCR amplification (Figure 4). The sequences are listed in the key resources table.
    **CRITICAL:** PAM sequences are excluded from the oligonucleotide sequences.
    ***Note:*** The length of the oligonucleotides is equal to or less than 60 nt, and they could be ordered using the regular oligo synthesis options.
    ***Optional:*** Use File S1 (pAAV 3×gRNA KO designing sheet.xlsm) for the oligonucleotide design (Figure 5). This Excel sheet allows semi-automatic design of oligonucleotide sequence (requires the use of a macro). Make sure to open this file with Excel software; otherwise, the macro does not work properly, and the sequence to order will not be generated properly. This step is optional, and manual oligonucleotide design yields the same outcome. However, manual insertion of reverse complement sequences may introduce human error; this Excel sheet helps minimize such mistakes.

**Figure 4:**
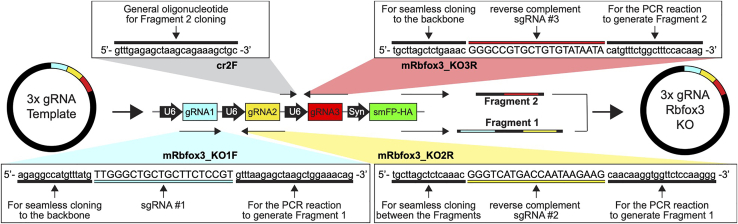
Oligonucleotide design for sgRNA cloning The sgRNA sequences without PAM are incorporated into the forward primer (mRbfox3_KO1F) and reverse primers (mRbfox3_KO2R and mRbfox3_KO3R), each containing additional overhangs for seamless cloning. PCR amplification of the 3× sgRNA Template (pAAV 3×gRNA KO; Template) using these primers generates Fragment 1 and Fragment 2. To target other genes, the blue, yellow, and red sequences should be replaced with the corresponding target-specific sequences. The primer cr2F is used universally in this reaction, regardless of the target sequence.

**Figure 5:**
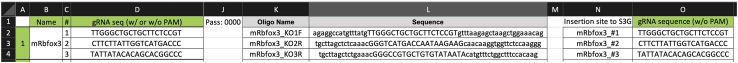
Semi-automatic design of oligonucleotides “pAAV 3×gRNA KO designing sheet.xlsm” facilitates semi-automatic design of oligonucleotides for ordering. While manually entering the target names and sgRNA sequences, the sheet automatically generates the oligonucleotide sequences for order. In Column B, enter the gene name. Column C is used to distinguish individual sgRNAs designed for the same target gene. For the first construct, numbers 1, 2, and 3 are typically used. If a second construct is designed for the same target gene, continue the numbering (e.g., 4, 5, 6) to differentiate between constructs. In Column D, enter the target sgRNA sequences. Sequences can be entered with or without the PAM. Oligonucleotide names and sequences for ordering will be automatically generated in Columns K and L. If PAM sequences are included in Column D, they are automatically excluded from the oligonucleotide sequences. The sgRNA sequences without PAM are displayed in Column O, regardless of whether the PAM sequences are present or not in Column D. Note: Most cells in the Excel file are locked to prevent accidental modification. If changes are required, the sheet can be unlocked via Review -> Unprotect Sheet using the password “0000” (not recommended).
  e. Download and open the ‘pAAV 3×gRNA KO; Template’ plasmid (Addgene, plasmid #240310) file in Benchling or Snapgene. Replace the sequences of sgRNA #1, #2, and #3 with the target sgRNA sequences (Figure 6).

**Figure 6:**
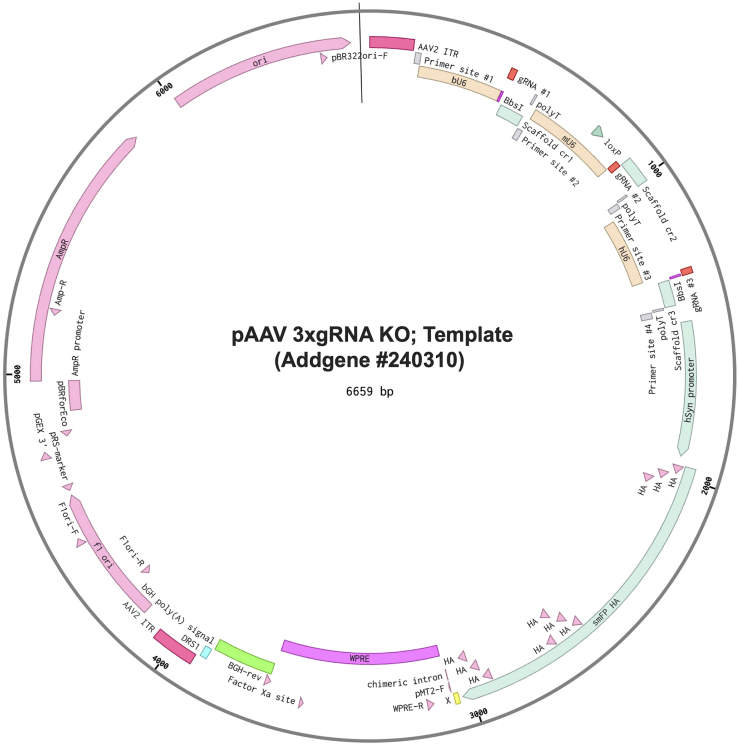
Schematic of CRISPR KO vector design and key elements bU6: Bovine U6 promoter, amplified from the pMJ114 plasmid (Addgene plasmid #85995; a gift from Jonathan Weissman), drives robust expression of the sgRNA in mammalian cells. mU6: Mouse U6 promoter, amplified from the pMJ179 plasmid (Addgene plasmid # 85996; a gift from Jonathan Weissman), drives robust expression of the sgRNA in mammalian cells. The loxP site in the mU6 was included in the original Addgene plasmid and was not used in this protocol. hU6: Human U6 promoter, amplified from the pMJ117 plasmid (Addgene plasmid #85997; a gift from Jonathan Weissman), drives robust expression of the sgRNA in mammalian cells. BbsI: There are two sites cleaved by the BbsI restriction enzyme to allow insertion of the 3× sgRNA sequences of interest. Scaffold cr1: Codon-optimized sgRNA scaffold sequence, amplified from the pMJ114 plasmid (Addgene plasmid #85995; a gift from Jonathan Weissman), forms the structural component of the sgRNA and facilitates Cas9 binding. Scaffold cr2: Codon-optimized sgRNA scaffold sequence, amplified from the pMJ179 plasmid (Addgene plasmid #85996; a gift from Jonathan Weissman), forms the structural component of the sgRNA and facilitates Cas9 binding. Scaffold cr3: Codon-optimized sgRNA scaffold sequence, amplified from the pMJ117 plasmid (Addgene plasmid #85997; a gift from Jonathan Weissman), forms the structural component of the sgRNA and facilitates Cas9 binding. Primer sites #1, 2, 3, and 4 are sequences designed to prepare oligonucleotides for Sanger sequencing or partial amplification of the backbone if necessary. hSyn: Human synapsin promoter, amplified from the pAAV-hSyn-EGFP plasmid (Addgene plasmid #50465; a gift from Bryan Roth), drives robust expression of the smFP-HA in neuronal cells. smFP-HA: GFP fused to 10× HA tags and is non-fluorescent due to mutations in the fluorophore. It enables visualization of the transduced cells, amplified from the pCAG_smFP HA plasmid (Addgene plasmid #59759; gift from Loren Looger). X: Sequence containing multiple stop codons.
2. CRISPR knockout plasmid construction.
  Here, we describe the procedures for assembling a triple sgRNA CRISPR/Cas9 knockout plasmid using a one-step cloning strategy.
  a. Fragment amplification.
    i. Amplify the ‘pAAV 3×gRNA KO; Template’ plasmid (Addgene, plasmid #240310) using the ordered oligonucleotides by PCR.

**Table:** PCR reaction mixture for amplifying Fragment 1 (467 bp)

‘pAAV 3×gRNA KO; template’ plasmid	20	ng
20 μM Forward primer: mRbfox3_KO1F	0.4	μl
20 μM Reverse primer: mRbfox3_KO2R	0.4	μl
CloneAmp HiFi PCR Premix	10	μl
MilliQ	up to 20	μl
Total	20	μl

**Table:** PCR reaction mixture for amplifying Fragment 2 (431 bp)

‘pAAV 3×gRNA KO; template’ plasmid	20	ng
20 μM Forward primer: cr2F	0.4	μl
20 μM Reverse primer: mRbfox3_KO3R	0.4	μl
CloneAmp HiFi PCR Premix	10	μl
MilliQ	up to 20	μl
Total	20	μl

**Table:** PCR cycling conditions

	Temperature	Time	Cycle
Initial Denaturation	98°C	1 min	1
Denaturation	98°C	10 s	35
Annealing	55°C	15 s	
Extension	72°C	45 s	
Final Extension	72°C	30 s	1
Hold	16°C	hold indefinitely
      **CRITICAL:** When you target the other genes of interest, replace the primers of KO1F, KO2R, and KO3R, and perform the same process. The cr2F is a general oligonucleotide for Fragment 2 cloning. Use a high-fidelity polymerase such as CloneAmp HiFi PCR Premix to minimize mutations.
      ***Note:*** This PCR reaction is typically robust regardless of the target sequence because (1) the primer-binding regions of the oligonucleotides are distinct from the sgRNA sequence regions, and (2) sgRNAs with higher MIT scores generally have optimal GC content and other sequence features that are compatible with efficient PCR amplification.
    ii. Run the PCR products on a 1.5% agarose gel, cut the bands, extract the PCR products using the kits, such as Nucleospin Gel PCR, and check the concentration of the products (Fragments 1 and 2) using the Nanodrop.
      **CRITICAL:** The concentration of the PCR product is usually over 10 ng/μL. If not, repeat the PCR amplification step.
      ***Note:*** See troubleshooting problem 1.
      **Pause point:** Extracted DNA can be stored at −20°C for over one year.
  b. Backbone digestion.
    i. Digest 1 μg of the ‘pAAV 3×gRNA KO; Template’ plasmid (Addgene, plasmid # 240310) using the BbsI restriction enzyme.

**Table:** Restriction enzyme reaction mixture

‘pAAV 3×gRNA KO; template’ plasmid	1	ug
BbsI-HF restriction enzyme	1	μL
rCutSmart Buffer	1	μL
MilliQ	up to 30	μL
Total	30	μL

**Table:** Restriction enzyme reaction conditions

	Temperature	Time
Restriction enzyme reaction	37°C	1–3 h
Hold	16°C	hold indefinitely
    ii. Run the digested product on a 0.7% agarose gel, cut the band, extract the product using the kits, such as Nucleospin Gel PCR, and check the concentration of the product using the Nanodrop.
      ***Note:*** The concentration of the digested plasmid is usually over 10 ng/μL. If not, repeat the digestion step.
      **Pause point:** Extracted DNA can be stored at −20°C for over one year.
  c. Plasmid assembly.
    i. Clone the PCR products into the digested plasmid backbone using the modified In-Fusion cloning protocol (https://www.takarabio.com/documents/User%20Manual/In/In-Fusion%20Snap%20Assembly%20User%20Manual.pdf).

**Table:** In-Fusion reaction mixture

BbsI digested plasmid (10 ng/ul)	1	μl
Fragment 1 (>10–80 ng/ul)	0.5	μl
Fragment 2 (>10–80 ng/ul)	0.5	μl
5x In-Fusion HD Enzyme Premix	0.5	μl
Total	2.5	μl

**Table:** In-Fusion cloning conditions

	Temperature	Time
In-Fusion reaction	50°C	15 min
Hold	16°C	hold indefinitely
    ii. Transform using NEB stable competent cells using the modified NEB stable transformation protocol (https://www.neb.com/en-us/protocols/5-minute-transformation-protocol-c3040).

**Table:** Plasmid transformation mixture

In-fusion reaction mixture	2.5	μl
NEB stable bacteria	10–15	μl
Total	12.5–17.5	μl

**Table:** Plasmid transformation condition

	Temperature	Time
Incubation before heat shock	4°C	10–15 min
Heat shock	42°C	30 s
Incubation after heat shock	4°C	10–15 min
      ***Note:*** AAV vector plasmids contain repeat ITR sequences, and NEB Stable E. coli strains are used to minimize recombination during transformation. However, other recombination-deficient E. coli strains are also suitable (e.g., One Shot Stbl3 chemically competent E. coli from Thermo Fisher).
    iii. Plate the bacteria on a 10 cm dish with LB agar with Ampicillin (100 μg/mL).
    iv. Incubate the plate at 37°C for 16–24 h.
3. CRISPR knockout plasmid verification.
  Here, we describe the procedures for validating correctly assembled triple sgRNA CRISPR/Cas9 knockout plasmids by colony PCR and sequencing.
  a. Colony PCR.
    i. The next day, pick individual colonies and perform colony PCR using the following conditions (Figure 7A),

**Table:** PCR reaction for colony PCR (584 bp)

Single colony	–	–
20 μM Forward primer: cr2F	0.4	μl
20 μM Reverse primer: hSyn 0R	0.4	μl
OneTaq 2x Master Mix	10	μl
MilliQ	up to 20	μl
Total	20	μl

**Table:** PCR cycling conditions

	Temperature	Time	Cycle
Initial Denaturation	94°C	5 min	1
Denaturation	94°C	30 s	30
Annealing	60°C	30 s	
Extension	68°C	30 s	
Final Extension	68°C	5 s	1
Hold	16°C	hold indefinitely

**Figure 7:**
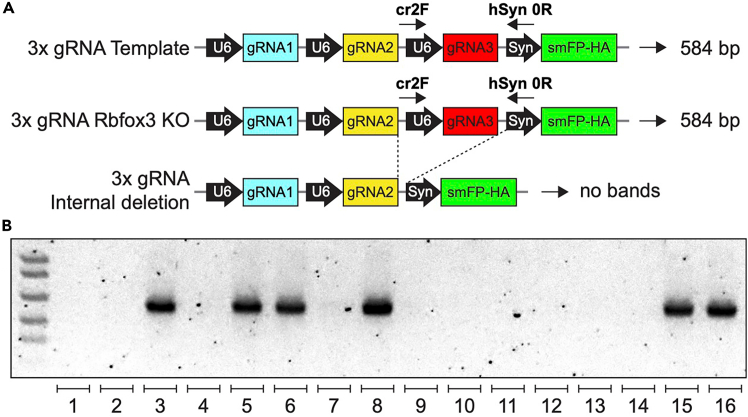
Colony PCR validation of 3× sgRNA insertion (A) Schematic of colony PCR using primers cr2F and hSyn 0R. Amplification of the 3× sgRNA template or correctly assembled 3× sgRNA *Rbfox3* knockout plasmid yields a 584 bp product, whereas plasmids with internal deletions do not produce a product. (B) Representative agarose gel showing colony PCR results. Bands at 584 bp indicate positive colonies.
      **CRITICAL:** 30 PCR cycles are optimal for detecting positive colonies while minimizing false positives; however, this may vary depending on the polymerase and PCR instrument used. Optimal conditions should be determined for each laboratory.
    ii. Run the PCR products on a 1.5% agarose gel.
      ***Note:*** Correctly assembled positive clones will show a 584 bp band, whereas negative colonies containing internal deletions will show no band (Figure 7B).
      ***Note:*** Internal deletions typically remove the sequence from downstream of gRNA2 through the gRNA3 region, resulting in loss of the hSyn 0R primer binding site (Figure 7A). The template plasmid produces a band of the same size; however, when backbone digestion is performed correctly, the template plasmid should not appear after cloning.
      ***Note:*** See troubleshooting problems 2 and 3.
  b. Plasmid purification.
    i. Pick the positive colonies and inoculate them into 8 mL of LB broth supplemented with ampicillin (100 μg/mL).
      **CRITICAL:** Pick and culture at least two independent colonies to ensure correct plasmid assembly.
      **Pause point:** Plates containing bacterial colonies can be stored at 4°C for over one week when sealed with Parafilm to prevent drying.
    ii. Incubate at 37°C for 16–24 h with shaking (250–300 rpm).
    iii. Isolate plasmid DNA from the entire 8 mL cultures using the NucleoSpin Plasmid Transfection-grade Miniprep Kit.
      **CRITICAL:** If the miniprep product is used directly for AAV production, a kit compatible with transfection-grade plasmid preparation should be used. Otherwise, any standard miniprep kit can be used, and the plasmids intended for viral production should be further purified using a midiprep kit or an equivalent method.
    iv. Sequencing the purified plasmid by Sanger sequencing to confirm the presence and accuracy of the sgRNA sequences using the following primers,
         hSyn 0R: cctggtcctaaaacccacttgcac
         mU6 R: caacaaggtggttctccaaggg.
      Alternatively, whole-plasmid sequencing services can be used.
      ***Note:*** Once the sequence is confirmed, the plasmids are ready for use in AAV vector production. The final plasmid generated in this protocol is referred to as ‘pAAV 3×gRNA KO; *Rbfox3*’, and is available from Addgene (plasmid #240311). The ‘pAAV 3×gRNA KO; Template’ could be used as a control that contains three non-targeting sgRNAs.
      ***Note:*** For the AAV production, vector packaging can be outsourced to commercial providers such as VectorBuilder or follow the established protocols.^1^
      ***Note:*** PHP.eB or PHP.S capsids are used for AAV vector packaging. PHP.eB is typically used for brain transduction via retro-orbital injection, whereas PHP.S is used for peripheral neuron transduction. However, with the intracerebroventricular injection described in this protocol, both serotypes achieve high transduction efficiency in brain neurons.

### Transduction and detection of CRISPR knockout by immunofluorescence

**Timing: 2 months**

**Timing: 1 day (step 4)**

**Timing: 1–3 days (step 5)**

**Timing: 2–3 days (step 6)**

Here, we describe neonatal AAV delivery into Cas9 knock-in mice and immunofluorescence-based validation of target protein loss in brain sections.

***Note:*** This step outlines the procedures for evaluating CRISPR-mediated knockout of the genes of interest in the mouse brain by the injection of AAV vectors. Cas9 knock-in mice are maintained in breeding cages, and AAV vectors are injected into neonatal pups at postnatal days 0–2 (P0–P2). This approach eliminates the need for precise embryonic staging and sacrifice of the pregnant mouse, enabling the procedure to be performed routinely and reproducibly. Immunofluorescence analysis is used to visualize the loss of target protein expression. We recommend waiting at least 3 weeks after AAV vector injection before tissue fixation, with more than 4 weeks preferred to achieve full knockout efficiency. In our in vitro experiments, nearly complete knockout is typically observed by 3 weeks post-infection, whereas knockout efficiency at 2 weeks does not reach maximal levels.

4. Animal preparation and AAV vector injection.
  Here, we describe neonatal intracerebroventricular AAV injection in Cas9 knock-in mice.
  ***Note:*** We describe the procedures for neonatal intracerebroventricular (ICV) AAV vector injection in Cas9 knock-in mice. The protocol below presents an example for *Rbfox3* knockout, but this protocol can be applied to the knockout of any other gene of interest.
  For visual guidance of the injection procedure, readers may refer to the JoVE video by Kim et al.^7^
  a. Set up mating cages for Cas9 knock-in mice.
    ***Note:*** We use the mice from Jax: 027650, but other spCas9 knock-in mice are theoretically compatible.
  b. Perform AAV vector injection in neonatal pups between postnatal day 0 and 2 (P0–P2) (Figure 8A). Both male and female mice were used in this study.

**Figure 8:**
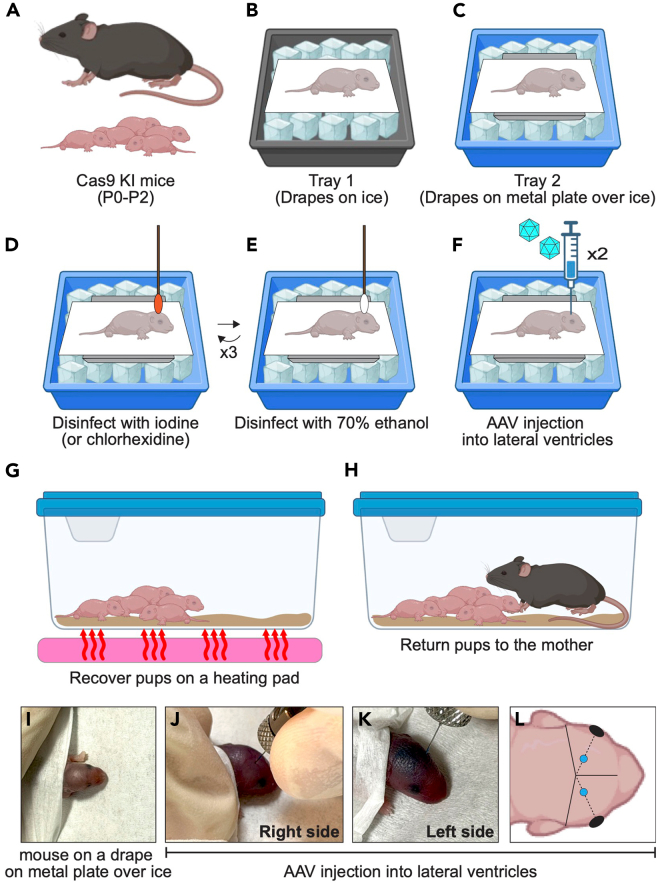
Neonatal AAV vector injection workflow for intracerebroventricular (ICV) injection (A) Neonatal Cas9 knock-in pups (P0–P2). (B) Cryoanesthesia on ice. (C) Transfer to a metal plate over ice. (D and E) Head disinfection with iodine and 70% ethanol. (F) AAV vector injection into the lateral ventricles. (G) Recovery on a heating pad. (H) Return of pups to the mother. (I–K) Representative images of the injection procedure. (L) Schematic of injection coordinates for ICV injection. The injection site (blue) is located approximately two-fifths of the distance from the lambda to the eye. Images are created with Biorender.com.
  c. Place neonatal pups on a tray with a sterile drape positioned directly on ice to induce cryoanesthesia (approximately 5 to 10 min) until the pups become pale and unresponsive to stimulation (Figure 8B).
  d. Transfer pups to another tray with a sterile drape placed on a metal plate over ice to maintain stable cooling during the procedure (Figures 8C and 8I).
  e. Disinfect the head of the pup using a cotton swab soaked in iodine or 4% chlorhexidine (Figure 8D).
  f. Disinfect the head with a cotton swab soaked in 70% ethanol (Figure 8E).
  g. Repeat steps (e) and (f) 3 times.
  h. Load 1–2 μL of AAV vector solution into a Hamilton syringe fitted with a 32-gauge needle.
  i. Inject the AAV vector solution into one lateral ventricle (Figures 8F and 8J).
    **CRITICAL:** Perform the injection slowly (∼5 s per step), including needle insertion, solution injection, and withdrawal. The injection site is approximately two-fifths of the distance from the lambda to the eye, which is visible through the skin (Figure 8L). Insert the needle perpendicular to the skin surface to a depth of ∼3 mm. Marking a depth on the needle may be helpful.
  j. Repeat the injection in the contralateral ventricle (Figures 8F and 8K).
  k. After injection, place pups in a cage on a heating pad and allow them to recover until normal color and movement return (Figure 8G).
  l. Return pups to their home cage with the mother (Figure 8H).
    **CRITICAL:** Pups typically recover at a rate of >90%. If the recovery rate is poor, the injection procedure and anesthesia conditions should be carefully re-evaluated.
    ***Note:*** See troubleshooting problem 4.
5. Preparation for the brain staining.
  Here, we describe perfusion, brain fixation, cryoprotection, embedding, and sectioning for downstream immunostaining.
  **CRITICAL:** Once the mice reach the age of analysis, prepare the following setup. Sectioning methods can be modified depending on the experimental purpose (e.g., floating sections cut by a vibratome may also be used).
  a. Prepare a pump system for perfusion, running 1× PBS using a 23-gauge needle.
  b. Anesthetize or euthanize the mice for cardiac perfusion using approved methods in each animal protocol.
  c. Cut through the rib cage and fully expose the heart.
  d. Cut the right atrium.
  e. Immediately after the cut, insert the 23-gauge needle into the left ventricle, and turn on the pump system.
  f. After running 20 mL of 1× PBS (5 mL/min), stop the pump system.
    **CRITICAL:** A flow rate of 5 mL/min is optimal for adult mice. Higher flow rates may damage the vasculature or brain structures, whereas lower flow rates may result in insufficient perfusion.
    **CRITICAL:** PFA perfusion is not performed to avoid excessive fixation. Fixation conditions should be optimized specifically for each experiment.
  g. Cut the head of the mouse, and then take out the brain.
  h. Immerse the brain into the ice-cold 4% PFA and incubate at 4°C for 1 h.
    **CRITICAL:** 1 h of PFA fixation is optimal for most antibodies used in our laboratory. However, fixation conditions should be optimized specifically for experiments.
    ***Note:*** We prepare 5 mL of PFA per well in 12-well plates.
  i. After 1 h of incubation, carefully remove all the PFA from the well, then add another 5 mL of 20% sucrose.
  j. Incubate the brain at 4°C for 16–24 h.
  k. The next day, make sure that the brains are sunk to the bottom of the well. Cut the brains depending on the regions of interest for each experiment, and embed them into the OCT compound in a plastic or metal sample box.
  l. Prepare isopentane with dry ice, and put the brain sample into the liquid until the OCT becomes white and fixed.
  m. Store the frozen brain blocks at −80°C.
    **Pause point:** Frozen brain blocks can be stored at −80°C for over one year.
  n. Prepare coverslips coated with 1% gelatin.
    i. Add 100 mL of MilliQ water to a beaker and bring to a boil using a microwave.
    ii. Add 1 g of gelatin and mix thoroughly using a stirrer.
    iii. If the gelatin is not fully dissolved, briefly reheat the solution in the microwave and continue mixing until completely dissolved.
    iv. Allow the solution to cool to 20°C–26°C.
    v. Dip each coverslip (22× 22 mm square) into the gelatin solution.
      ***Note:*** Coverslips of other sizes could also be used.
    vi. Allow the coverslips to dry at 20°C–26°C for 16–24 h.
  o. Cut the brain sections using a cryostat.
    i. Prepare 20 μm sections.
      ***Note:*** Although thicker sections can be used, limited antibody penetration may result in uneven staining, depending on the antibodies. Conversely, thinner sections are more fragile during staining.
    ii. Take the brain sections onto a coverslip coated with 1% gelatin.
      ***Note:*** The gelatin-coated coverslips are prepared by the laboratory, are cost-effective, and are suitable for the routine immunostaining procedures demonstrated below.
      **Pause point:** Brain sections on coverslips can be stored at 4°C after mounting. However, staining should be performed as soon as possible and within 1 week, as longer storage may reduce staining quality.
6. Immunostaining.
  Here, we describe permeabilization, blocking, antibody staining, and mounting for imaging.
  **CRITICAL:** The volumes of solutions indicated are based on a single 22× 22 mm square coverslip. If coverslips of other sizes are used, the volumes should be adjusted accordingly.
  a. Remove the remaining OCT compound from the coverslips using fine forceps.
  b. Permeabilize and block the coverslips by incubating them in 200 μl of PBTGS buffer (see recipe in the “materials and equipment” section) at 20°C–26°C for 1 h.
    ***Note:*** We do the permeabilization and blocking steps at the same time.
  c. Remove the reagent and incubate the brain sections at 4°C for 16–24 h with primary antibodies diluted in 150–200 μl of PBTGS.
    **CRITICAL:** Antibody dilutions must be optimized for each antibody.
  d. The next day, wash the brain sections 3 times with 200–500 μl of PBTGS (5 min each).
  e. Remove the reagent and incubate the brain sections at 20°C–26°C for 2 h with secondary antibodies diluted in 150–200 μl of PBTGS.
    **CRITICAL:** Antibody dilutions must be optimized for each antibody.
    **CRITICAL:** Ensure all incubations with secondary antibodies are done in the dark to avoid photobleaching.
  f. Wash coverslips once with 200–500 μl of PBTGS, once with 200–500 μl of 0.1 M PB, and once with 200–500 μl of 0.05 M PB for 5 min each to remove unbound secondary antibodies.
  g. Mount the coverslips onto the microscope slides.
    i. Apply two drops of mounting medium per coverslip.
      ***Note:*** In this protocol, Vectashield plus (Vector Labs) anti-fade mounting medium was used.
    ii. Once the coverslips are settled onto the slide glass, put a paper towel on it and put a weight (we usually use a tube rack, e.g., Fisher, #22-313630) on the slide glass.
    iii. Incubate at 20°C–26°C for 10 min in the dark. This step removes excess amount of the mounting medium between the coverslip and the slide glass.
      **CRITICAL:** Do not put too much weight. It will cause too much reduction of the mounting medium, resulting in the inclusion of air bubbles around the brain sample.
    iv. After removing excess mounting medium, seal the edges of the coverslip with nail polish to prevent movement.
    v. Carefully wipe and clean the surface of coverslips with ethanol and DI water.
  h. Acquire images using a microscope.
    **CRITICAL:** Imaging parameters (e.g., objective, exposure time, gain, and laser power) should be optimized depending on the target structure, antibody, and imaging system used.
    ***Note:*** See troubleshooting problems 5, 6, and 7.

## Expected outcomes

**Figure 9:**
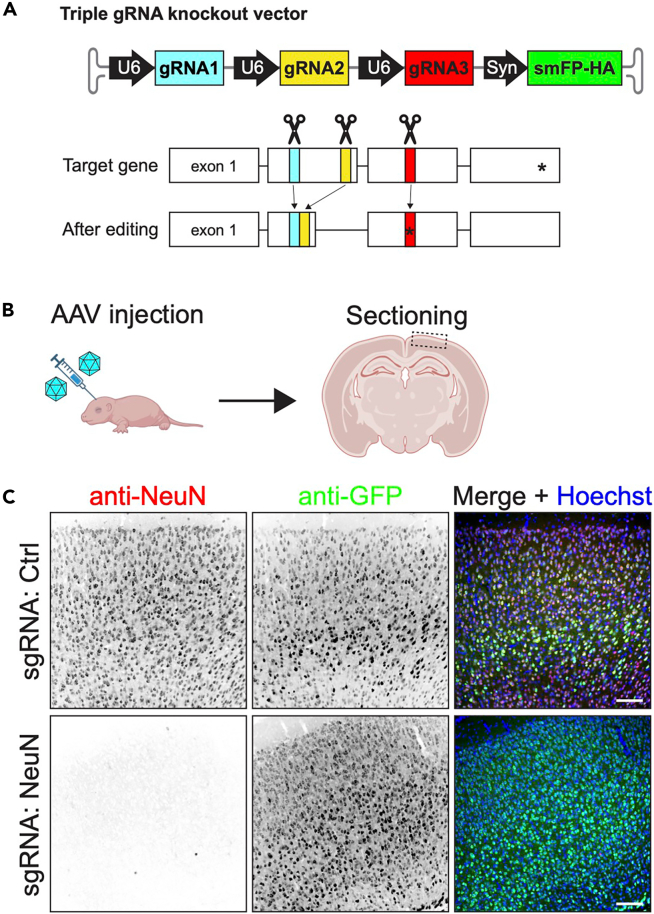
Rbfox3 (NeuN) knockout in the mouse brain (A) Schematic representation of the AAV-based triple sgRNA knockout vector and simultaneous targeting of three independent regions within the gene of interest. Deletion and premature stop codons are generated in the target genome. (B) Schematic illustration of AAV vector injection into neonatal mouse pups followed by brain sectioning. The viral concentration of gRNA control was 2.51E+11. GC/ml and sgRNA NeuN was 3.73E+11 GC/ml. Images are created with Biorender.com. (C) Immunofluorescence staining of brain sections using antibodies against NeuN and GFP. Nuclei are counterstained with Hoechst. Scale bars, 100 μm.

The AAV-based triple single guide RNA (3× sgRNA) knockout vector system described here has been validated across multiple target genes in vitro and in vivo.^1^^,^^8^^,^^9^^,^^10^ Using the one-step cloning workflow, multiple plasmids can be generated in parallel. The success rate of correctly assembled colonies is typically ∼50%, with approximately half of the colonies exhibiting internal deletions. Therefore, screening multiple colonies by colony PCR is essential. Those plasmids could be packaged into AAV vectors and used to transduce target cells or tissues (Figures 9A and 9B). When plasmid construction, AAV vector production, and transduction are successful, this approach typically yields high knockout efficiency (Figure 9C). Based on our experience, knockout efficiencies typically range from ∼60% to >90% in well-transduced tissues, although this may vary depending on the target gene, sgRNA performance, and AAV vector transduction efficiency. When reliable antibodies are available, we prioritize immunofluorescence-based validation, although western blotting can also be used. When suitable antibodies are not available, RNA-based methods such as fluorescence in situ hybridization (FISH) or RNAscope, as well as PCR-based detection of genomic DNA alterations, can be used as alternative approaches. Successful knockout is indicated by a substantial reduction or loss of the target protein signal in transduced cells (Figure 9C). It is first important to confirm that the signal of the transduction marker–smFP-HA–is robustly detectable under control conditions. smFP-HA is a GFP fused to 10x HA tags and is non-fluorescent due to mutations in the fluorophore. Immunostaining with anti-HA antibodies yields strong signals in transduced neurons. Alternatively, most anti-GFP antibodies can also be used for detection.

One commonly observed pattern is mosaic loss of signal, in which a subset of cells shows complete loss of staining while neighboring cells retain a strong signal. This pattern typically reflects variability in AAV vector transduction efficiency and can often be improved by increasing viral titer. However, for studies focusing on cell morphology or cell-autonomous phenotypes, mosaic knockout is not necessarily problematic, provided that a sufficient number of fully knockout cells are present.

For quantitative analysis, knockout efficiency can be evaluated by: (1) calculating the percentage of cells lacking detectable signal (cell-based quantification), or (2) measuring overall fluorescence intensity reduction across the tissue (intensity-based quantification) using ImageJ or other equivalent software. Cell-based quantification is generally preferred when mosaicism is present, whereas intensity-based analysis is suitable for samples with a high proportion of knockout cells. Thresholds for defining knockout may vary depending on experimental conditions and should be carefully determined.

To validate results for sgRNA-specific effects, we recommend designing two independent sets of sgRNAs targeting distinct regions of the gene. In our experiments, we typically compare control, knockout construct 1 (KO1), and KO2 in parallel. Ideally, both sgRNA sets should yield comparable phenotypes, strengthening confidence in on-target effects. If no qualitative or quantitative differences are observed between control and knockout conditions, this may indicate insufficient sgRNA activity. In such cases, comparison between sgRNA sets (KO1 and KO2) is critical. If substantial differences are observed between sgRNA sets, additional sgRNA sets should be designed and tested to confirm the phenotype. It is also important to consider potential antibody-related issues, such as background signal or cross-reactivity.

## Limitations

This protocol requires sufficient Cas9 expression in the target cell. In vivo experiments described here rely on spCas9 knock-in mice to achieve robust editing efficiency. Although AAV-mediated delivery of spCas9 can be used effectively in cultured cells, high-efficiency knockout in vivo requires mice that constitutively express spCas9.

AAV vector transduction and CRISPR-mediated genome editing can result in mosaicism, meaning that not all transduced cells will be genome edited. While using three sgRNAs increases the probability of successful bi-allelic genome editing, incomplete editing may still occur in a subset of transduced cells. Together, knockout efficiency is highly dependent on sgRNA sequence, AAV vector quality, viral titer, and injection accuracy. This can be mitigated by optimizing viral titer, improving injection conditions, and selecting highly efficient sgRNAs.

The 3× sgRNA knockout vector system uses a neuron-specific hSyn promoter, which restricts expression of the marker gene (smFP-HA) to neuronal cells. However, the sgRNAs are expressed from U6 promoters, which are not cell-type specific. Therefore, if the detection of a marker gene expression is not required, this approach may not be strictly restricted to neurons.

Finally, validation using a single monoclonal antibody only confirms loss of the specific epitope, not necessarily the entire protein. The use of polyclonal antibodies or antibodies targeting multiple regions is recommended. Complementary validation at the RNA or DNA level is also recommended.

## Troubleshooting

### Problem 1 (related to one-step cloning, step 2a)

No or weak PCR product for Fragment 1 or 2.

### Potential solutions

• Verify the oligonucleotide sequences. Use of the supplemental Excel sheet is recommended to minimize design errors.
• Use a fresh, high-quality template plasmid and a high-fidelity polymerase (e.g., CloneAmp HiFi).
• Optimize PCR conditions, including annealing temperature, cycle number, and extension time, according to the polymerase manufacturer’s recommendations.

### Problem 2 (related to one-step cloning, step 3a)

No positive colonies.

### Potential solutions

• Confirm complete BbsI digestion of the backbone by agarose gel electrophoresis and purify the fully linearized backbone.
• Gel-purify PCR fragments to remove primer dimers and nonspecific amplification products.
• Verify the concentration of the digested backbone and PCR fragments before performing the In-Fusion reaction.

### Problem 3 (related to one-step cloning, step 3a)

Internal deletions between U6 cassettes were detected by colony PCR, or mutations in sgRNA cassettes or elsewhere in the plasmid.

### Potential solutions

• Screen multiple colonies (five or more) to identify correctly assembled clones.
• Use recombination-deficient bacterial strains, such as NEB Stable, to minimize rearrangements.
• Perform plasmid sequencing for at least two independent clones to confirm sequence integrity.

### Problem 4 (related to transduction and detection, step 4)

Poor or inconsistent AAV vector delivery into the brain.

### Potential solutions

• Add a dye such as Fast Green (final concentration 0.1%) or Trypan Blue (final concentration 0.05%) to the injection solution to visualize delivery.
• In neonatal mice at P0, successful injection can be confirmed by observing the spread of the solution within the lateral ventricles through the head. This visualization is typically not possible at later stages at P2.
• If most of the injected solution comes back from the head of the injection site, the injection parameters are likely not optimal. Optimize injection angle, position, and depth. However, leakage of the solution between the skull and skin shortly after injection is relatively common and does not necessarily indicate failed delivery.

### Problem 5 (related to transduction and detection, step 6)

Low knockout efficiency after AAV vector injection.

### Potential solutions

• Verify AAV vector titer and quality prior to injection.
• Confirm successful transduction by immunostaining for overexpressed smFP-HA using anti-HA or anti-GFP antibodies to assess expression in target cells.
• Allow sufficient time post-injection (>3–4 weeks) before tissue analysis to ensure maximal editing efficiency.
• Consider that knockout of certain genes may be lethal in pups. In such cases, successful gene disruption may lead to loss of affected animals, resulting in an apparent reduction in knockout efficiency. This may be mitigated by restricting gene disruption to specific brain regions through stereotaxic injection at later developmental stages.

### Problem 6 (related to transduction and detection, step 6)

High background or poor immunostaining signal.

### Potential solutions

• Optimize the immunostaining protocol, including adjustment of primary and secondary antibody dilutions and blocking conditions.
• Use anti-fade mounting medium and protect samples from light during and after staining.
• Confirm that fixation time and conditions are optimal for the staining.

### Problem 7 (related to transduction and detection, step 6)

Observed knockout phenotypes do not align with expected results.

### Potential solutions

• Although selecting sgRNAs with high MIT specificity scores reduces off-target effects, unintended editing may still occur. Validate observed phenotypes using an independent set of sgRNAs targeting the same gene.
• Confirm protein loss by other methods (e.g., western blotting or RT-PCR).
• Consider potential biological compensation or incomplete editing in mosaic tissues.

## Resource availability

### Lead contact

Further information and requests for resources and reagents should be directed to the lead contact, Yuki Ogawa (yuki.ogawa@sc.edu).

### Technical contact

Technical questions on executing this protocol should be directed to the technical contact, Yuki Ogawa (yuki.ogawa@sc.edu).

### Materials availability

All reagents generated in this study are available from the lead contact with a completed material transfer agreement.

### Data and code availability

All data reported in this paper will be shared by the lead contact upon request. All original code is available in this paper’s supplemental information (File S1). Any additional information required to reanalyze the data reported in this paper is available from the lead contact upon request.

## Acknowledgments

This work was supported by startup funds from the 10.13039/100005912University of South Carolina to Y.O. We thank Kayla Otto, Alyssa Schelble, Jianyu Gan, and Duc V.M. Nguyen for helpful discussions.

## Author contributions

Y.O. conceived the study. G.D. and Y.O. wrote the original draft, created the figures, and prepared the final manuscript. Y.O. conducted the biochemical and immunofluorescence experiments and analyses. Y.O. developed the CRISPR tools, handled AAV vector production, performed animal injections, and conducted brain immunostaining. All authors reviewed and approved the final manuscript.

## Declaration of interests

The authors declare no competing interests.

## Declaration of generative AI and AI-assisted technologies in the writing process

During the preparation of this work, the authors used ChatGPT to improve the grammar and readability of the manuscript. After using this tool/service, the authors reviewed and edited the content as needed and take full responsibility for the content of the publication.

## Supplemental information

File S1. pAAV 3xgRNA KO designing sheet

## References

[1] Ogawa Y., Nguyen D.V.M., Ogawa A., Rasband M.N. (2025). Hide-and-Seek genome editing reveals that Gephyrin is required for axo-axonic synapse assembly. Proc. Natl. Acad. Sci. USA.

[2] Hsu P.D., Lander E.S., Zhang F. (2014). Development and applications of CRISPR-Cas9 for genome engineering. Cell.

[3] Swiech L., Heidenreich M., Banerjee A., Habib N., Li Y., Trombetta J., Sur M., Zhang F. (2015). In vivo interrogation of gene function in the mammalian brain using CRISPR-Cas9. Nat. Biotechnol..

[4] Pacesa M., Pelea O., Jinek M. (2024). Past, present, and future of CRISPR genome editing technologies. Cell.

[5] Sunagawa G.A., Sumiyama K., Ukai-Tadenuma M., Perrin D., Fujishima H., Ukai H., Nishimura O., Shi S., Ohno R.I., Narumi R. (2016). Mammalian Reverse Genetics without Crossing Reveals Nr3a as a Short-Sleeper Gene. Cell Rep..

[6] Adamson B., Norman T.M., Jost M., Cho M.Y., Nuñez J.K., Chen Y., Villalta J.E., Gilbert L.A., Horlbeck M.A., Hein M.Y. (2016). A Multiplexed Single-Cell CRISPR Screening Platform Enables Systematic Dissection of the Unfolded Protein Response. Cell.

[7] Kim J.Y., Grunke S.D., Levites Y., Golde T.E., Jankowsky J.L. (2014). Intracerebroventricular viral injection of the neonatal mouse brain for persistent and widespread neuronal transduction. J. Vis. Exp..

[8] Melton A.J., Palfini V.L., Ogawa Y., Oses Prieto J.A., Vainshtein A., Burlingame A.L., Peles E., Rasband M.N. (2024). TRIM46 Is Required for Microtubule Fasciculation In Vivo But Not Axon Specification or Axon Initial Segment Formation. J. Neurosci..

[9] Ogawa Y., Lim B.C., George S., Oses-Prieto J.A., Rasband J.M., Eshed-Eisenbach Y., Hamdan H., Nair S., Boato F., Peles E. (2023). Antibody-directed extracellular proximity biotinylation reveals that Contactin-1 regulates axo-axonic innervation of axon initial segments. Nat. Commun..

[10] Zhang W., Fu Y., Peng L., Ogawa Y., Ding X., Rasband A., Zhou X., Shelly M., Rasband M.N., Zou P. (2023). Immunoproximity biotinylation reveals the axon initial segment proteome. Nat. Commun..

